# Electroencephalogram Mechanism of Dexmedetomidine Deepening Sevoflurane Anesthesia

**DOI:** 10.3389/fnins.2022.913042

**Published:** 2022-05-12

**Authors:** Lei Zhang, Hua Li, Liyun Deng, Kun Fang, Yuanyuan Cao, Cheng Huang, Erwei Gu, Jun Li

**Affiliations:** ^1^Inflammation and Immune Mediated Diseases Laboratory of Anhui Province, School of Pharmacy, Anhui Institute of Innovative Drugs, Anhui Medical University, Hefei, China; ^2^Department of Anesthesiology, The First Affiliated Hospital of Anhui Medical University, Hefei, China; ^3^Key Laboratory of Anesthesiology and Perioperative Medicine of Anhui Higher Education Institutes, Anhui Medical University, Hefei, China; ^4^Key Laboratory of Anti-inflammatory and Immune Medicine, Ministry of Education, Hefei, China; ^5^Institute for Liver Diseases of Anhui Medical University, Hefei, China

**Keywords:** dexmedetomidine, cognitive function, sevoflurane, electroencephalogram, anesthesia depth

## Abstract

Dexmedetomidine, as an α2-adrenoceptor agonist, plays anti-sympathetic, sedative and analgesic roles in perioperative period. Also, dexmedetomidine can reduce the minimal alveolar concentration (MAC) of sevoflurane and the risk of postoperative cognitive dysfunction (POCD) induced by sevoflurane anesthesia. But so far, the electroencephalogram (EEG) mechanism of dexmedetomidine deepening sevoflurane anesthesia is not clear. In this study, by analyzing the changes of the power spectrum and bicoherence spectrum of EEG before and after dexmedetomidine infusion, the EEG mechanism of dexmedetomidine deepening sevoflurane anesthesia was studied. We analyzed dexmedetomidine-induced changes in power spectrum and bicoherence spectrum in 23 patients under sevoflurane anesthesia. After anesthesia induction, the sevoflurane concentration was maintained at 0.8 MAC for 15 min, and then dexmedetomidine was administered at a loading dose of 0.8 μg/kg in 10 min, followed by a maintenance rate of 0.5 μg⋅kg^–1^⋅h^–1^. Frontal EEG data from 5 min before and 10 min after dexmedetomidine infusion were compared. After dexmedetomidine infusion, the mean α power peak decreased from 6.09 to 5.43 dB and shifted to a lower frequency, the mean θ bicoherence peak increased from 29.57 to 41.25% and shifted to a lower frequency, and the median α bicoherence peak increased from 41.49 to 46.36% and shifted to a lower frequency. These results demonstrate that dexmedetomidine deepens sevoflurane anesthesia, and enhances α and θ bicoherences while shifting peak values of these bands to lower frequencies through regulating thalamo-cortical reverberation networks probably.

## Introduction

Dexmedetomidine is an active dextral isomer of medetomidine, which plays anti-sympathetic, sedative, and analgesic roles by activating the α2-adrenoceptor. It is commonly used as a sedative and anesthetic adjunct during perioperative period and can reduce postoperative neurological complications under sevoflurane anesthesia (Gerresheim and Schwemmer, 2013; Keating, 2015). In practice, the minimal alveolar concentration (MAC) is often used to evaluate the potency of various inhalational anesthetics, and as an indicator of anesthesia depth (Eger et al., 1965). Several studies have shown that dexmedetomidine can reduce the MAC and requirement of sevoflurane (Gozalo-Marcilla et al., 2013; Patel et al., 2013). However, the electroencephalographic (EEG) mechanism of the effect of dexmedetomidine on sevoflurane anesthesia remains unclear.

Spontaneous EEG activity is a commonly used physiological index reflecting the state of consciousness or depth of anesthesia (Freye and Levy, 2005). A growing body of evidence suggests that anesthetics could induce characteristic oscillations by altering or disrupting information processing and communications in the brain (Chauvette et al., 2011; Purdon et al., 2013; Akeju et al., 2014b). These anesthesia-induced oscillations are different as they act on different targets (Musizza and Ribaric, 2010; Purdon et al., 2015b). Dexmedetomidine primarily acts at presynaptic α2 adrenergic receptors to hyperpolarize locus coeruleus neurons by decreasing norepinephrine release (Nacif-Coelho et al., 1994). The reduced release of norepinephrine can result in loss of inhibitory inputs to the preoptic area of the hypothalamus, loss of excitatory inputs to intralaminar nucleus of the thalamus, cortex and decreased thalamo-cortical connectivity (Brown et al., 2011; Akeju et al., 2014a). Sedation under dexmedetomidine is characterized in EEG by spindles and slow-δ oscillations (Huupponen et al., 2008; Xi et al., 2018). Sevoflurane exerts sedative and anesthetic effects mainly through binding at multiple targets in central nervous system including gamma-aminobutyric acid type A receptors (GABA_A_R), N-methyl-D-aspartic acid receptors (NMDAR), and so on (Nishikawa, 2004). Akeju et al. (2014b) have found that sevoflurane induces coherent frontal α oscillations and slow-wave oscillations to sustain the unconscious state, indicating that sevoflurane may interfere with thalamo-cortical information processing and fragment cortical activity. In view of this, the combination of dexmedetomidine and sevoflurane may produce synergistic or enhancing effects on sedation levels and EEG performance.

Researches continue to validate that α and δ-θ oscillations in EEG induced by anesthetics are associated with neural network regulation and resonance of the thalamo-cortical and cortico-thalamic axons (Schneider and Kochs, 2007; Ching et al., 2010; Akeju et al., 2014b). Bicoherence analysis is a power-independent bispectral analysis that has been developed to detect cross-frequency phase coupling and examine non-linear regulation of brain electrical activities (Hayashi et al., 2008b). In non-linear reverberating system such as seen between the cortex and thalamus, the output signal from the reverberation circuit is reenter into the system as the input signal, leading to self-regulation characteristics and quadratic phase coupling of a variety of different signal wave components (Hayashi et al., 2008b; Araki et al., 2018). Several studies have indicated that bicoherence can reveal the reverberating components and evaluate the electroencephalographic mechanism of combined use of anesthetics (Hagihira et al., 2002; Morimoto et al., 2006; Araki et al., 2018).

In this study, we analyzed changes in EEG bicoherence resulting from dexmedetomidine infusion, and studied the EEG mechanism underlying the effect of dexmedetomidine on sevoflurane anesthesia. We hypothesized that dexmedetomidine deepens sevoflurane anesthesia, and that it changes the power spectra and bicoherence patterns by regulating thalamo-cortical networks.

## Materials and Methods

Ethical approval for this study (PJ2019-14-17) was provided by the Ethics Committee of the First Affiliated Hospital of Anhui Medical University, Hefei, China (Chairperson Prof. Heng Wang) on November 1, 2019. Written informed consent was obtained from all patients. The trial was registered before patient enrollment at http://www.ChiCTR.org.cn (ChiCTR1900026955).

### Study Population

**FIGURE 1:**
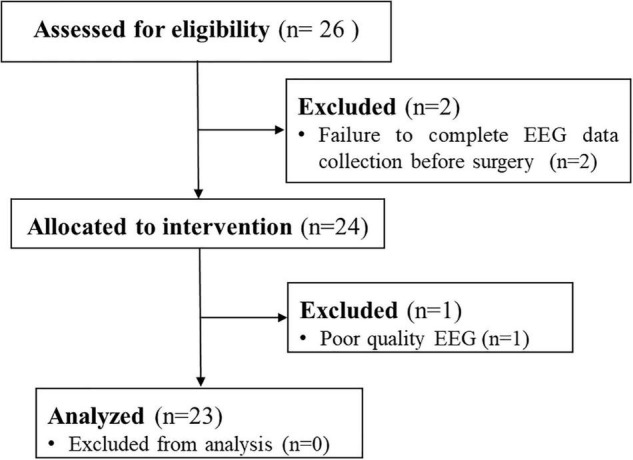
Flow chart of patient selection and exclusion.

Patients aged 18–65 years with American Society of Anesthesiologists (ASA) physical status 1 and 2 who underwent non-cranial and non-cardiac surgeries were recruited. Patients with dementia, intellectual disability or other neuropsychiatric disorders, severe bradycardia, histories of cerebrovascular disorders, hearing impairment or other factors lead to communication difficulty and those receiving treatment with α2 agonists or antagonists were excluded. Of the 26 enrolled patients, two cases were excluded because EEG data collection was not completed before surgery commenced in accordance with our protocol, one case was excluded due to poor quality EEG. So, twenty-three patients were included in the final analysis. Figure 1 presents a flow chart of patient selection and exclusion.

### Anesthesia Methods

Patients fasted for at least 8 h before surgery and were given no preoperative medication. Standard vital signs (i.e., of non-invasive blood pressure, electrocardiogram, end-tidal carbon dioxide, and pulse oxygen saturation) and EEG monitoring were initiated upon patients’ entry into the operating room, and the baseline vital signs were recorded.

Anesthesia was induced with a combination of sevoflurane (6%), sufentanil (0.5 μg/kg), and cisatracurium (0.2 mg/kg). All patients underwent laryngeal mask ventilation, and the sevoflurane concentration was maintained at 0.8 MAC for anesthesia maintenance. After approximately 15 min of stable sevoflurane maintenance (Morimoto et al., 2006), dexmedetomidine (0.8 μg/kg) was administered in a 10 min intravenous infusion. The dose of dexmedetomidine was then changed to 0.5 μg⋅kg^–1^⋅h^–1^ for continuous infusion (Harsoor et al., 2014). We maintained intraoperative blood pressure and heart rate fluctuating within 20% of base values. Drugs such as atropine, noradrenaline and ephedrine were used as needed.

### Data Collection and Electroencephalogram Preprocessing

Data collection for this study was completed before surgery began. Frontal EEG data were recorded continuously by a SedLine brain function monitor (Masimo Corporation, Irvine, CA, United States) with a sampling rate of 178 Hz and a preamplifier bandwidth of 0.5–89 Hz, using the system’s standard Sedtrace electrode array [six electrodes at approximately Fp1, Fp2, F7, F8, Fpz (ground), and 1 cm above Fpz (reference)]. Electrode impedance for each channel was ≤5 kΩ.

The patient state index (PSI) is a clinically validated measure of monitoring depth of anesthesia and sedation. PSI is a dimensionless number in the range between 100 (fully awake) and 0 (deeply anesthetized) with decreasing values indicating increasing levels of hypnosis (Drover and Ortega, 2006; Buget et al., 2016). The 95% spectral edge frequency (SEF95) is one of the processed EEG measures and the values typically range between 0 and 30 Hz with decreasing values indicating a lower level of responsiveness (Tonner and Bein, 2006). The PSI and SEF95 were recorded every minute.

We applied the linear finite impulse response filter from the EEGLAB toolbox to the raw EEG signals (0.5–50 Hz) (Tort et al., 2008). An experienced investigator visually excluded noise and artifacts. For each subject, we selected two 3-min-long artifact-free EEG segments at 5 min before and 10 min after dexmedetomidine infusion for spectral and bicoherence analysis.

### Spectral Analysis

We examined power spectra, defined as quantifications of EEG power at each frequency, and constructed spectrograms, consisting of temporally consecutive power spectra, using the multi-taper method with the Chronux toolbox (Percival and Walden, 1993). The following parameters were used for spectral analysis: window length = 2 s with 0 s overlap, time-bandwidth product = 3, number of tapers = 5 and spectral resolution = 3 Hz. Group-level spectrograms were constructed for the two timepoints by taking the medians across all patients. Group-level spectra and 95% confidence intervals (CIs) were computed by taking median across spectrograms at each timepoint by bootstrap method (Purdon et al., 2015a). Briefly, we resampled spectrogram estimates to obtain replicates and calculated bootstrap group median spectra. We also calculated differences between the group median spectra estimates from the two timepoints for each frequency. The differences were considered significant only when the contiguous frequency bandwidth exceeded the spectral resolution (Akeju et al., 2016). These procedures were repeated 10,000 times, and the percentile method was used to calculate 95% CIs.

### Bicoherence Analysis

Similar to the methods used in previous studies (Hayashi et al., 2008a,b, 2010, 2014; Araki et al., 2018), bicoherence at the two timepoints was examined by calculating all pairs of frequencies from 0.5 to 20 Hz at 0.5-Hz intervals, which were represented as two-dimensional moving averages. Nine points of bicoherence were used to calculate diagonal bicoherence (every 0.5 Hz from 1.5 to 20 Hz). The 3-min-long EEG signals were divided into 360 2-s epochs, with 75% overlap, and the Blackman window function was applied. The following equations were used to calculate bicoherence:

Sum of the absolute triple product $\left[\mathrm{sTP}\left(f_{1},f_{2}\right)\right]=\sum_{\text{i}=1}^{\text{L}}\left|{\text{X}}_{\text{i}}\left(f_{1}\right)X_{\text{i}}\left(f_{2}\right){\text{x}}_{\text{i}}\text{*}\left(f_{1}+f_{2}\right)\right|$

Bispectrum $\left[B\left(f_{1},f_{2}\right)\right]=\left|\sum_{\text{i}=1}^{\text{L}}{\text{X}}_{\text{i}}\left(f_{1}\right)X_{\text{i}}\left(f_{2}\right){\text{x}}_{\text{i}}\text{*}\left(f_{1}+f_{2}\right)\right|$ and

Bicoherence $\text{BIC}\left({\text{f}}_{1},{\text{f}}_{2}\right)=100\frac{\text{B}\left({\text{f}}_{1},{\text{f}}_{2}\right)}{\text{sTP}\left({\text{f}}_{1},{\text{f}}_{2}\right)}$,

Where j is the epoch number, X_j_ (f_1_) is a complex value calculated by Fourier transformation of the jth epoch and X_i_* (f_1_+f_2_) is the conjugate of X_i_ (f_1_+f_2_). A bicoherent spectrum was represented along the diagonals (the same frequency pairs). The group median diagonal bicoherence (f1 = f2) and 95% CIs were calculated using a bootstrap procedure, with medians of bootstrap samples drawn from the full sample of diagonal bicoherence for each subject at each timepoint. MATLAB was used for all bootstrap calculations (Araki et al., 2018). This procedure was repeated 10,000 times, and the percentile method was used to calculate 95% CIs.

### Statistical Analysis

The PSI, SEF95, α power and bicoherence peaks, frequencies at those peaks, and the θ bicoherence peak and their frequencies values from electroencephalogram were compared between 5 min before and 10 min after dexmedetomidine infusion under sevoflurane anesthesia. According to the results of Shapiro–Wilk normality tests of the difference between the two timepoints, the Wilcoxon signed-rank test (non-normal parameters) or paired *t*-test (normal parameters) were used. The GraphPad Prism software version 5.0 was used for the statistical analysis, and the results were expressed as medians (25th and 75th percentiles) or means ± SD, respectively. Mean/median difference and 95% CIs between groups were calculated by the bootstrap method. *P* < 0.05 was considered to represent statistically significance.

## Results

### Basic Information

The demographic and clinical characteristics of the 23 patients who participated in this study are presented in Table 1.

**TABLE 1:** Characteristics of patients receiving dexmedetomidine infusions under sevoflurane anesthesia.

Characteristic	Mean (SD) or *n* (%)
Age (years)	45.00 (6.68)
Sex (male)	9 (39.13%)
Weight (kg)	58.50 (8.70)
Height (m)	1.62 (0.05)
BMI (kg/m^2^)	23.65 (3.07)
**ASA physical status**	
I	9 (39.13%)
II	14 (60.87%)

*BMI, Body Mass Index.*

### Representative Time Courses of Power and Bicoherence Spectra

**FIGURE 2:**
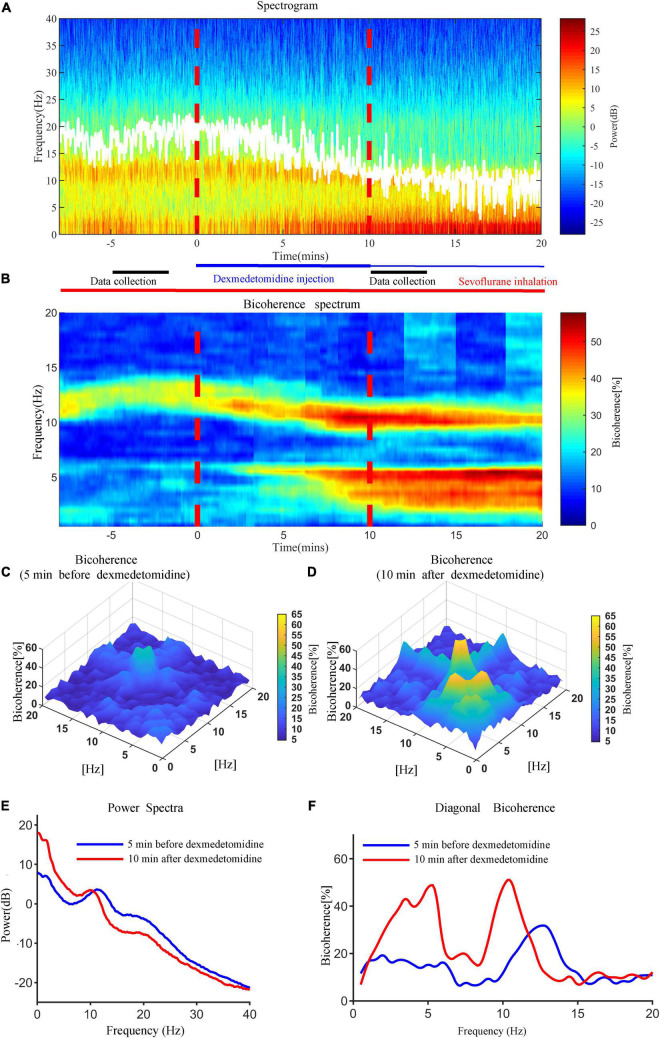
Representative time courses of power and bicoherence spectra 5 min before and 10 min after dexmedetomidine infusion for a 29-year-old man under sevoflurane anesthesia. **(A)** Time-frequency spectrogram of the frontal cortex during anesthesia. **(B)** Bicoherence spectrum of the frontal cortex during anesthesia. White solid lines in **(A)** represent SEF95. **(C)** Bicoherence spectra 5 min before dexmedetomidine infusion for all pairs of frequencies. **(D)** Bicoherence spectra 10 min after dexmedetomidine infusion for all pairs of frequencies. **(E)** Power spectra 5 min before (blue line) and 10 min after (red line) dexmedetomidine infusion. **(F)** Diagonal bicoherence 5 min before (blue line) and 10 min after (red line) dexmedetomidine infusion.

Representative time courses of power and bicoherence spectra before and after dexmedetomidine infusion for a 29-year-old man undergoing meniscoplasty under sevoflurane anesthesia are shown in Figure 2. 10 min after dexmedetomidine infusion, EEG activity changed gradually and then achieved the maximum effect; the peak value of θ-band bicoherence increased and moved to a lower frequency, the α-band power peak decreased and moved to a lower frequency, and the bicoherence peak increased and moved to a lower frequency.

### The Comparison of Patient State Index and Spectral Edge Frequency 95

**FIGURE 3:**
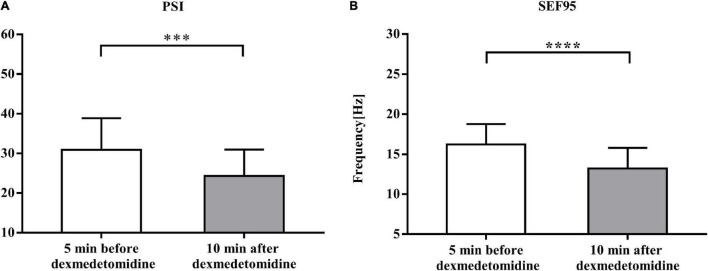
Comparison of EEG monitoring data 5 min before and 10 min after dexmedetomidine infusion. **(A)** Comparison of values of patient status index (PSI) data 5 min before and 10 min after dexmedetomidine infusion under sevoflurane anesthesia. Data are given as mean ± SD (Paired *t-*test probability is indicated as: ^***^P < 0.001; ****P < 0.0001, *n* = 23). **(B)** Comparison of values of spectral edge frequency 95 (SEF95) data. Details as in **(A)**.

Compared with baseline (5 min before dexmedetomidine infusion), the SEF95 and PSI decreased after dexmedetomidine infusion [from 16.24 ± 2.54 Hz to 13.22 ± 2.58 Hz (*P* < 0.0001) and from 30.91 ± 7.98 to 24.30 ± 6.68 (*P* < 0.001), respectively] (Figure 3).

### Group-Level Spectrograms and Bicoherence Analysis

**FIGURE 4:**
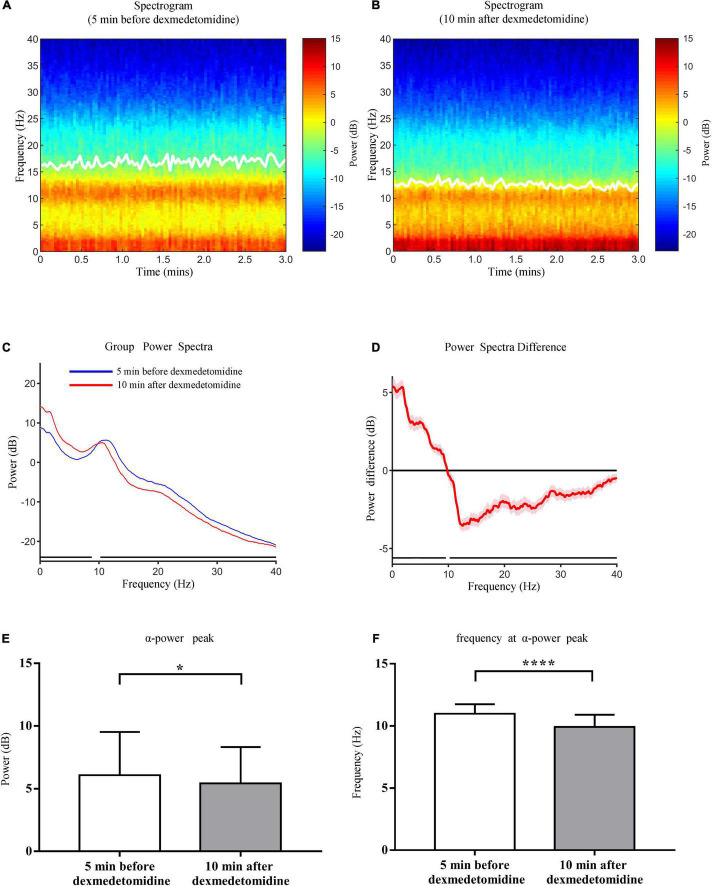
Comparison of group-level power spectral analysis 5 min before and 10 min after dexmedetomidine infusion. **(A)** Frontal spectrogram of 23 cases 5 min before dexmedetomidine infusion. **(B)** Frontal spectrogram 10 min after dexmedetomidine infusion. White solid lines in **(A,B)** represent SEF95. **(C)** Comparison of group-level power spectra 5 min before (blue line) and 10 min after (red line) dexmedetomidine infusion, shading represents 95% CI range. **(D)** Median spectral power difference of two periods at each frequency, shading represents 95% CI range. The horizontal black lines in **(C,D)** represent frequency segments with significant differences across two time periods (0–9.56 Hz and 10.22–40 Hz). **(E)** Comparison of power of α peaks 5 min before and 10 min after dexmedetomidine infusion. Data are given as mean ± SD (Paired *t-*test probability is indicated as: *P < 0.05; ****P < 0.0001, *n* = 23). **(F)** Comparison of frequencies of α peaks. Details as in **(E)**.

**FIGURE 5:**
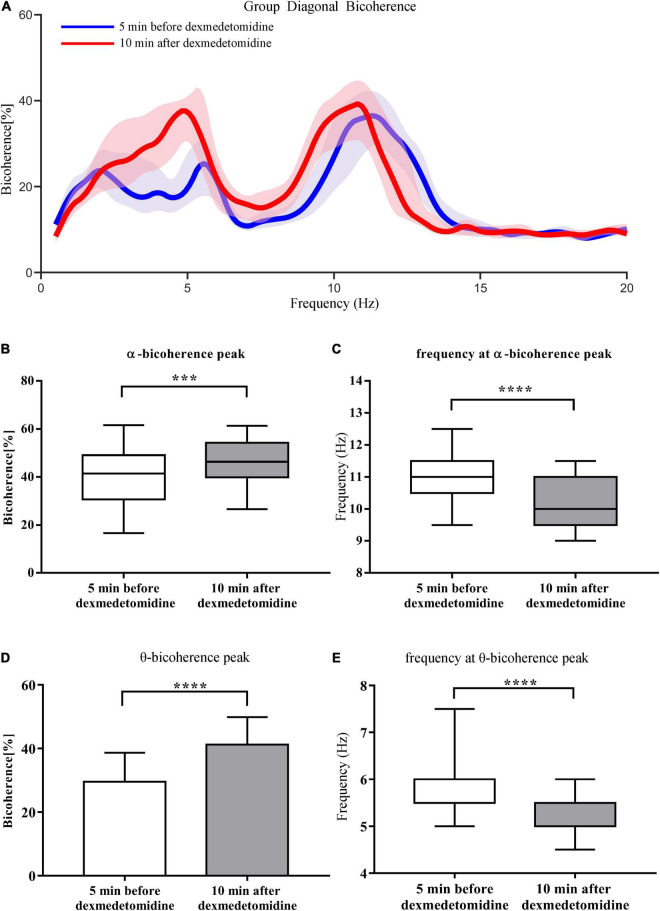
Comparison of group-level diagonal bicoherence analysis 5 min before and 10 min after dexmedetomidine infusion. **(A)** Comparison of diagonal bicoherence spectra from 23 cases 5 min before (blue line) and 10 min after (red line) dexmedetomidine infusion, shading represents 95% CI range. **(B)** Comparison of bicoherence of α peaks 5 min before and 10 min after dexmedetomidine infusion. Data are given as box plots (Wilcoxon signed-rank test probability is indicated as: ***P < 0.001; ****P < 0.0001, *n* = 23). **(C)** Comparison of frequencies of α bicoherence peaks. Details as in **(B)**. **(D)** Comparison of bicoherence of θ peaks. Data are given as mean ± SD (Paired *t-*test probability is indicated as: ****P < 0.0001, *n* = 23). **(E)** Comparison of frequencies of θ bicoherence peaks. Details as in **(B)**.

Group-level spectrograms show that slow-wave power increased after dexmedetomidine infusion (Figures 4A,B). The α power peaks decreased [from 6.09 ± 3.45 dB to 5.43 ± 2.90 dB (*P* < 0.05); bootstrap mean difference, −0.66 (−2.46 to 1.16) dB] and moved to lower frequencies [from 10.98 ± 0.78 Hz to 9.92 ± 1.00 Hz (*P* < 0.0001); bootstrap mean difference, −1.06 (−1.56 to −0.56) Hz (Figure 4)]. After dexmedetomidine infusion, the θ-band bicoherence peaks increased and moved to lower frequencies [from 29.57 ± 9.14% to 41.25 ± 8.67% (*P* < 0.0001), bootstrap mean difference 11.63% (6.42–16.50%) and from 5.50 (5.50, 6.00) Hz to 5.00 (5.00, 5.50) Hz (*P* < 0.0001), bootstrap median difference –0.5 (–0.5 to 0) Hz (Figures 5A,D,E)]. The same pattern was observed for the α-band bicoherence peaks [from 41.49% (30.68%, 49.08%) to 46.36% (39.89%, 54.21%) (*P* < 0.001), bootstrap median difference 4.83% (−2.55 to 14.11%) and from 11.00 (10.50, 11.50) Hz to 10.00 (9.50, 11.00) Hz (*P* < 0.0001), bootstrap median difference –1.00 (–1.50 to 0.00) Hz (Figures 5B,C)].

## Discussion

The findings of the present study showed that significant decreases in both PSI and SEF95 values after intravenous dexmedetomidine infusion in patients under sevoflurane anesthesia, indicating dexmedetomidine can induce sevoflurane anesthesia from moderate level (PSI value approximately 31) into a deeper level (PSI value approximately 24). After dexmedetomidine infusion, the α power peak decreased and moved to a lower frequency, and the θ and α bicoherence peaks increased and moved to lower frequencies. Previous studies have found that coherent α and θ oscillations are generally viewed as originating primarily from thalamo-cortical oscillations (Morimoto et al., 2006; Araki et al., 2018). These results indicate that dexmedetomidine augments the effect of sevoflurane anesthesia probably by regulating thalamo-cortical networks.

### Neural and Electroencephalogram Mechanisms Behind Spectrograms and Bicoherence Changes

When dexmedetomidine is administered as a low-dose infusion, the EEG shows a combination of slow-δ oscillations with spindles (Huupponen et al., 2008). As the infusion rate of dexmedetomidine increases, the spindles will disappear and the power of slow-δ oscillations will increase. The spindles induced by dexmedetomidine are thought to be generated by thalamo-cortical loop mechanisms (Brown et al., 2011). The slow-wave oscillations induced by dexmedetomidine probably result from decreased excitatory inputs to the cortex and decreased adrenergically mediated excitatory inputs to the basal forebrain, the intralaminar nucleus of the thalamus and cortex (Brown et al., 2011). Similarly, different sevoflurane concentrations can cause different EEG manifestations. When sevoflurane is administered at sub-MAC concentrations, the EEG shows slow-δ oscillations and coherent α oscillations, as the concentration is increased to MAC levels and above, a strong θ oscillation appears, indicates a more profound state of unconsciousness (Akeju et al., 2014b; Purdon et al., 2015b). The EEG changes induced by sevoflurane indicate decreasing frontal and thalamo-cortical connectivity (Ranft et al., 2016). In the current study, we found that after dexmedetomidine infusion under sub-MAC sevoflurane anesthesia, the α power peaks decreased and moved to lower frequencies, and the slow-wave power increased, suggest that dexmedetomidine enhances sevoflurane anesthesia and may be associated with decreased thalamo-cortical connectivity.

The use of bicoherence analysis, as in this study, enables quantification of the degree of phase coupling between signal components and the elucidation of EEG features that cannot be analyzed using simple power spectra (Sigl and Chamoun, 1994; Hayashi et al., 2008b). The signal processing techniques used in previous studies enable the evaluation only of linear processes, thereby ignoring potential non-linear interactions between signal components (Hayashi et al., 2008a). Bicoherence analysis can track changes in non-linear re-input systems, such as that between the cortex and thalamus (Hayashi et al., 2008b). Researchers have reported that when sevoflurane concentration increase from 1% to 3%, α and δ-θ peak frequencies decrease proportionally, and bicoherence in the δ-θ area increases with deepening anesthesia, indicating the obtained features are consistent with characteristics of the thalamo-cortical reverberating networks (Hayashi et al., 2008b). Consistent with their results, we found that α and θ bicoherence increased and their peak frequencies moved to a lower frequency after dexmedetomidine infusion, suggesting dexmedetomidine deepen sevoflurane anesthesia partly by regulating the thalamo-cortical reverberation networks.

### Potential Molecular Mechanism of Dexmedetomidine Deepening Sevoflurane Anesthesia

Dexmedetomidine is a highly selective α2-adrenoceptor agonist, which has strong sedative and analgesic effects (Gerresheim and Schwemmer, 2013). α2-adrenoceptors are seven-fold transmembrane receptors belonging to the G-protein–coupled receptor family (Kamibayashi and Maze, 2000). Postsynaptic α2-adrenoceptors exist in many tissues, such as the cerebral cortex and thalamus. Dexmedetomidine activates the G_i_ protein after interacting with the α2-adrenoceptor (Khan et al., 1999), inhibiting the activity of adenylate cyclase (Afonso and Reis, 2012; Gu et al., 2015; Im et al., 2018). Adenylate cyclase catalyzes the formation of cyclic AMP (cAMP), and cAMP can activate downstream signals as an important second messenger molecule by acting on membrane ion channels. Dexmedetomidine inhibits the voltage-gated sodium channel current by reducing the amount of cAMP, this may be the mechanism by which it deepens sevoflurane anesthesia (Nelson et al., 2001; Gu et al., 2015). The G_i_ protein also activates potassium ion channels (Khan et al., 1999; Chen et al., 2009), causing cell hyperpolarization, which reduces the activation of excitable cells in the central nervous system, inhibits the discharge of locus neurons (Kamibayashi and Maze, 2000) and inhibits the activity of the norepinephrine pathway. So, dexmedetomidine might regulate the thalamo-cortical reverberation networks through G_i_-related mechanisms.

### Limitations

There are several limitations in this study. First, the theoretical analysis of the results is based on our extrapolation of the elucidated electrophysiological knowledge, we did not study the brain nuclei associated with the bicoherence and power spectrum changes, or their possible internal linkages or molecular mechanisms. In future, we could use functional magnetic resonance imaging and animal experiments to identify the specific pathways and networks involved in dexmedetomidine-induced deepening of sevoflurane anesthesia. Second, we only assessed frontal EEG, the bicoherence spectrums may differ between different cortical areas (Hayashi et al., 2014), we will use high-density electroencephalogram to explore a more comprehensive mechanism in the future. Third, we did not examine the effect of different doses of dexmedetomidine on sevoflurane anesthesia, which may have yielded different electroencephalogram and molecular mechanisms.

## Conclusion

After dexmedetomidine infusion during sevoflurane anesthesia, the PSI and SEF95 decreased, the α power peak decreased and moved to a lower frequency, and the θ and α bicoherence peaks increased and moved to lower frequencies. These results revealed the EEG mechanisms on dexmedetomidine-induced deepening of sevoflurane anesthesia, which might through regulating thalamo-cortical reverberation networks.

## Data Availability Statement

The original contributions presented in the study are included in the article/supplementary material, further inquiries can be directed to the corresponding author/s.

## Ethics Statement

The studies involving human participants were reviewed and approved by the Ethics Committee of the First Affiliated Hospital of Anhui Medical University. The patients/participants provided their written informed consent to participate in this study.

## Author Contributions

LZ conceived the project, supervised the data analysis, and wrote and revised the manuscript. HL collected the data and wrote the manuscript. LD analyzed the data and wrote the manuscript. KF collected and analyzed the data. YC and CH revised the manuscript. EG and JL designed the project, wrote and revised the manuscript. All authors contributed to the article and approved the submitted version.

## Conflict of Interest

The authors declare that the research was conducted in the absence of any commercial or financial relationships that could be construed as a potential conflict of interest.

## Publisher’s Note

All claims expressed in this article are solely those of the authors and do not necessarily represent those of their affiliated organizations, or those of the publisher, the editors and the reviewers. Any product that may be evaluated in this article, or claim that may be made by its manufacturer, is not guaranteed or endorsed by the publisher.
